# Chasing the Cure Around the Globe: Medical Tourism for Cancer Care From Developing Countries

**DOI:** 10.1200/JGO.17.00087

**Published:** 2017-08-28

**Authors:** Humaid O. Al-Shamsi, Marwan Al-Hajeili, Sadir Alrawi

**Affiliations:** **Humaid O. Al-Shamsi**, University of Sharjah, Sharja; **Sadir Alrawi**, Alzahra Private Hospital, Dubai, United Arab Emirates; and **Marwan Al-Hajeili**, King Abdulaziz University, Jeddah, Saudi Arabia

## TO THE EDITOR:

The recent commentary by Benedetti et al^1^ in *Journal of Global Oncology* accurately identifies and describes the growing, underestimated, and under-researched area of medical tourism for cancer care^2^ and the associated ethical issues arising when patients seek care in international settings. Having been involved on both sides, as the accepting physicians during our practice in the United States and more recently as the referring physicians during our current practice in the United Arab Emirates (UAE) and Saudi Arabia, we would like to discuss and clarify some of the ethical and practical issues pertaining to medical tourism for cancer care in our countries.

Benedetti et al^1^ discussed medical tourism in which patients travel to less developed countries for medical care; however, they were hesitant to define the current practice of patients with cancer from developing countries traveling to developed countries as medical tourism. The definition of medical tourism is broad and simple: “People traveling abroad with the expressed purpose of accessing medical treatment.”^3(p410)^ This definition is not restricted by the direction of travel to or from developed countries. What is unique about medical tourism by patients traveling from less developed countries is that a significant number of these patients are sponsored by their governments, especially those from oil-rich countries. The UAE spent an estimated US$163 million in 2013 on medical tourism for cancer care.^4^

Benedetti et al^1^ speculated the reasons for medical tourism included second opinions, research, or specialized care that is unavailable in the patients’ home countries. From our experience, most patients are seeking second opinions rather than participation in clinical trials or specialized care lacking in their own countries. Because of a lack of official data in this regard, it is difficult to quantify the percentage of patients whose cancer care is indeed indicated abroad. We estimate less than 10% of such patients truly require specialized care abroad. This medical tourism results largely, as the authors indicated, from the patients’ and their families’ belief that newer technologies and better medicine exist abroad, and therefore, patients’ outcomes will be improved overseas.

Unfortunately, there is a significant misconception about the health care and cancer care available in the UAE and Saudi Arabia. For example, the health system in the UAE was ranked 27th in the world by the WHO in 2010.^5^ Many of the physicians practicing in these countries (including the authors of this letter) are American Board certified in their medical and surgical fields and have clinical and research experience in the United States; however, the patients, and largely their families, demand second opinions for cancer treatment that is available in their own country, despite the local physicians’ reassurances. This leads to another dilemma when providing medical reports to patients, because they demand their physicians state that the cancer treatment is not available locally, when in fact the care is available. This is to facilitate approval for their travel abroad for medical treatment. The approval process itself is complex and performed by different independent sponsoring agencies in the country. Patients may try different outlets and usually will accept the sponsoring agency that sponsors them first. Most cases get approved within 6 to 10 weeks, which is a long period, especially knowing that an additional 4 to 6 weeks will be required for the embassy to obtain the medical appointment abroad. The entire process from the date of diagnosis to the appointment date abroad can require 10 to 16 weeks, which can clearly be problematic when treating patients with cancer, whose treatment is time sensitive. Any significant delay in initiating the treatment plan will have a detrimental effect on a patient’s outcome. In addition, many cases are rejected by the sponsoring agencies within the country, because the treatments are available locally. However, in most cases, patients and their families request special exemptions, which can be approved in some instances. A patient whose efforts to travel abroad fail usually returns to the same physician and expresses dissatisfaction with his report, because he or she believes this was the cause of the rejection. In most cases, he or she also demands a referral to another physician to receive the treatment locally.

We agree with the recommendations of Benedetti et al^1^ to improve and standardize the patient intake process. However, direct communication with the referring providers and conversations with the patients before travel, as the authors recommended, remain broad and technically challenging for various and complex reasons; for example, the information available to the accepting physician and the quality of that information (eg, pathology), which can be critical and alter the entire diagnosis and management plan, may be limited. The form of communication (telephone call, video call), time zone differences, the willingness of the parties involved (the referring physician, the accepting physician, the patient, the patient’s family, the interpreter, and a representative from the sponsoring agency) to participate in such communication, and the form and process of reimbursement for this service make the authors’ recommendations for direct communication with the referring providers and conversations with the patients difficult to implement, impractical, and inconvenient for all parties involved.

Benedetti et al^1^ correctly identified that acceptance of international patients is vulnerable to a conflict of interest because of the financial gain to the accepting institution. We doubt that any institution that gains financially from these international patients will make changes to its existing policies about accepting these patients if these changes could adversely affect its revenue or business model. This gray area remains unclear and unresolved, and more independent research is required.

We agree with Benedetti et al^1^ that there are challenges in communicating with international patients, as a result of language as well as cultural differences. It is not uncommon for a patient’s family to request that the treating physician not disclose the cancer diagnosis to the patient. This is a challenging ethical situation and can cause conflict between the physician and the patient’s family if the physician insists on communicating with the patient about the details of his or her disease. Growing evidence in this field supports that most patients from Saudi Arabia (and we believe it also applies to patients from the neighboring UAE, which shares the same language, culture, and religion) prefer to be informed about their diagnosis and prognosis, despite their families’ protective requests to withhold the information.^6,7^ We agree with the authors’ recommendations that having a cultural navigator would likely improve and facilitate communication with international patients. We recommend a cultural navigator who shares the same language and culture, which could further facilitate the communication, especially in centers with a high volume of patients from these countries.

The quality of language interpretation is another issue that we would like to highlight. From our experience, the quality of interpretation varies by the interpreter, and the information conveyed by the interpreter can differ from the intended purpose of the treating team as a result of inaccurate or poor word choice. It is not uncommon for interpreters to include their personal views or recommendations, which can bias patients’ medical decisions. More research is needed in this area to clarify the effect of the quality of language interpretation on these patients’ care.

In conclusion, we agree that more research is needed to address these practical and ethical issues surrounding cancer care for international patients, especially from the sponsoring governments in collaboration with the accepting institutions abroad, to meet the intended purposes of the government-sponsored medical tourism programs and enhance the quality of care for these patients.
